# A reduced-dimensional polar hybrid perovskite for self-powered broad-spectrum photodetection†

**DOI:** 10.1039/d0sc06112c

**Published:** 2021-01-04

**Authors:** Dong Li, Wentao Wu, Shiguo Han, Xitao Liu, Yu Peng, Xiaoqi Li, Lina Li, Maochun Hong, Junhua Luo

**Affiliations:** State Key Laboratory of Structural Chemistry, Fujian Institute of Research on the Structure of Matter, Chinese Academy of Sciences Fuzhou Fujian 350002 China lilina@fjirsm.ac.cn; Fujian Science & Technology Innovation Laboratory for Optoelectronic Information of China Fuzhou Fujian 350108 P. R. China; University of Chinese Academy of Sciences Beijing 100049 China

## Abstract

Polar hybrid perovskites have been explored for self-powered photodetection benefitting from prominent transport of photo-induced carriers and the bulk photovoltaic effect (BPVE). However, these self-powered photodetection ranges are relatively narrow depending on their intrinsic wide bandgaps (>2.08 eV), and the realization of broad-spectrum self-powered photodetection is still a difficult task. Herein, we successfully obtained a polar multilayered perovskite, (I-BA)_2_(MA)_2_Pb_3_I_10_ (**IMP**, MA^+^ = methylammonium and I-BA^+^ = 4-iodobutylammonium), *via* rational dimension reduction of CH_3_NH_3_PbI_3_. It features the narrowest bandgap of 1.71 eV in a BPV material. As a consequence, the integration of narrow bandgap and BPVE causes the self-powered photodetection to extend to 724 nm for **IMP**, and a repeatable photovoltaic current reaching 1.0 μA cm^−2^ is acquired with a high “on/off” ratio of ∼10^3^ and photodetectivity (∼10^9^ Jones) at zero bias. This innovative research provides a foothold for adjusting the physical properties of hybrid perovskites and will expand their potential for self-powered broad-spectrum detection.

## Abstract 2

A polar hybrid perovskite with a wide-spectrum absorption extending to 724 nm was obtained . Benefitting from the narrow bandgap and bulk photovoltaic effects, self-powered broad-spectrum photodetection was achieved in hybrid perovskites.

## Graphical abstract

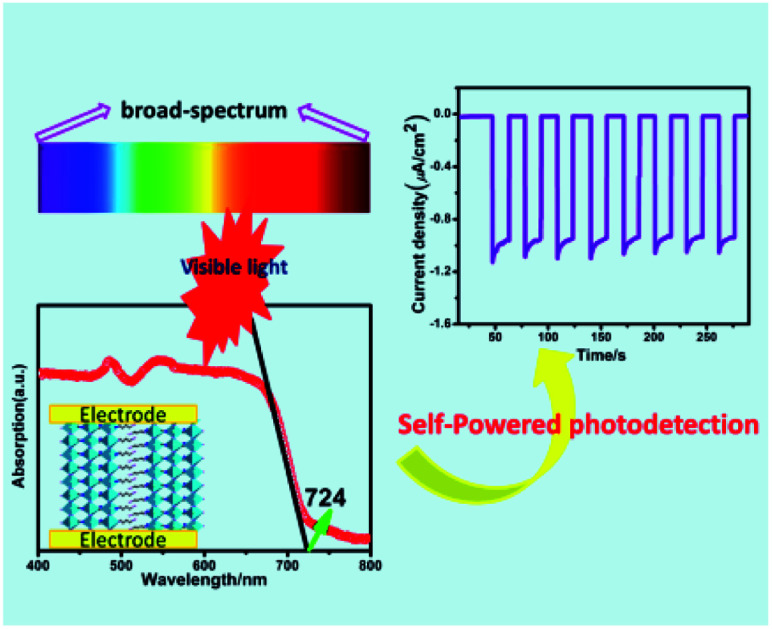

## Introduction

Self-powered photoelectric detection is of great significance to next-generation miniature and cost-effective photoelectric devices.^1–8^ Self-powered photodetection based on the bulk photovoltaic effect (BPVE) of polar materials has attracted intense attention recently.^9,10^ Compared to the traditional self-powered photodetectors constructed by p–n junctions or Schottky barriers,^11,12^ BPVE induced self-powered photodetection eliminates the complicated interface engineering and fabrication process.^13,14^ Notably, self-powered visible-blind ultraviolet photodetection depending on the BPVE has been realized in BaTiO_3_, (K_0.5_Na_0.5_)–(Mn_0.005_Nb_0.995_)O_3_, and La-doped Pb(Zr,Ti)O_3_.^15–17^ However, these inorganic oxides suffer from wide bandgaps (>2.7 eV) and low concentrations of photo-induced carriers, which limit their potential detection range. In this context, it is very intriguing and still challenging to acquire broad-spectrum candidates with the BPVE for high-performance self-powered photodetection.

Currently, three-dimensional (3D) CH_3_NH_3_PbI_3_ perovskite displaying broad-band absorption and high carrier mobility has shown great promise for solar cell and photodetection applications.^18–20^ But its high structural symmetry leads to the lack of BPVE. Owing to the fact that there is no built-in electric field to facilitate carrier separation and transport, it can only work well with an applied external power source or based on a p–i–n structure.^21–23^ Thus, the rational introduction of symmetry breaking to achieve the BPVE in hybrid perovskites is essential.^24,25^ Benefitting from the structural compatibility and tunability of hybrid perovskites, Xiong's group has designed low-symmetry polar two-dimensional (2D) hybrid perovskites, such as (4,4-difluorocyclohexylammonium)_2_PbI_4_ and [*R*- and *S*-1-(4-chlorophenyl)ethylammonium]_2_PbI_4_,^26,27^ by employing large organic cations. They feature interesting ferroelectricity with intrinsic BPVEs. Furthermore, the BPVE was successfully achieved in chiral-polar layered lead-iodide perovskites (*S*/*R*-MPA)_2_(MA)Pb_2_I_7_ designed *via* introducing chiral organic cations.^28^ However, they still possess relatively wide bandgaps (>2.08 eV). It is well known that the inorganic layer is one of the determinants of the bandgap of a hybrid perovskite,^29–33^ and thus a multilayered I-based perovskite with a polar structure is promising to acquire a narrow bandgap toward broad-spectrum self-powered photodetection. In addition, the increase of the inorganic layer is expected to benefit the transport of photo-induced carriers, which results in high performance photodetection.^34–36^

Here, we present a new Ruddlesden–Popper 2D hybrid perovskite, (I-BA)_2_(MA)_2_Pb_3_I_10_ (**IMP**, MA^+^ = methylammonium, and I-BA^+^ = 4-iodobutylammonium), which is fabricated by the dimension reduction of the 3D prototype hybrid perovskite CH_3_NH_3_PbI_3_. It is noteworthy that **IMP** exhibits a wide absorption extending to 724 nm, corresponding to a narrow optical bandgap of ∼1.71 eV, close to the 1.50 eV of CH_3_NH_3_PbI_3_.^37^ Moreover, the polar structure enables the BPVE in **IMP**. Taking advantage of these above distinct performances, self-powered broad-spectrum photodetection with a large zero-bias photocurrent of 1.0 μA cm^−2^ was obtained with a high “on/off” ratio of ∼10^3^ and photodetectivity (∼10^9^ Jones) at zero bias. To the best of our knowledge, **IMP** features the narrowest bandgap in BPV materials for promising self-powered photodetection.

## Results and discussion

### Structure description

**Fig. 1:**
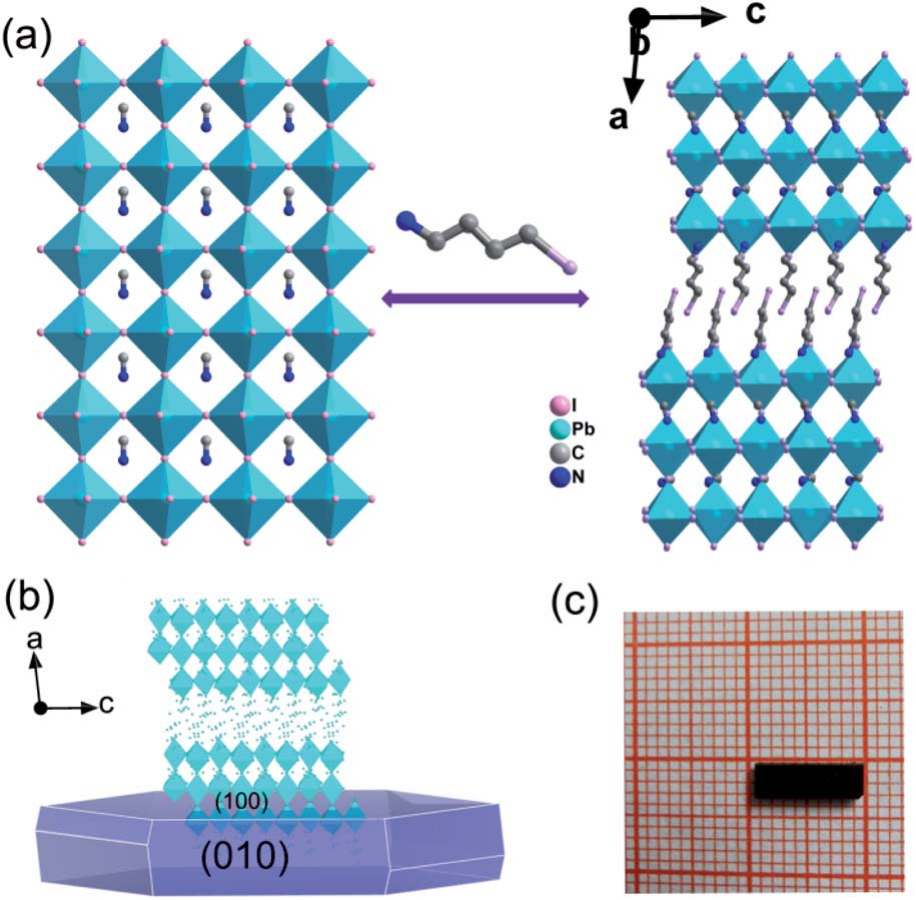
(a) Trimming three-dimensional CH_3_NH_3_PbI_3_ to design a 2D structure of **IMP** viewed along the *b*-axis. (b) Typical growth morphology for crystals of **IMP**. (c) Solution-grown bulk crystal of **IMP** with dimensions of 9.0 × 3.0 × 1.5 mm^3^.

Crystals of **IMP** were acquired from concentrated HI solution through a temperature cooling process. The measured PXRD patterns of the **IMP** are similar to those of the simulated results, which indicates that the synthesized **IMP** is a pure phase. Crystals exposed to the ambient atmosphere after a month present identical PXRD patterns to those of the original sample, revealing that **IMP** is environmentally stable (Fig. S1†). Single crystal X-ray diffraction reveals that **IMP** crystallizes in the monoclinic system with a polar space group of *Pc* at room temperature (Table S1†). The observed second harmonic generation further confirms the polar structure (Fig. S2†). As shown in Fig. 1a, **IMP** adopts a Ruddlesden–Popper 2D trilayered architecture with [Pb_3_I_10_]_∞_ inorganic sheets. The organic MA^+^ cations reside in the cavities formed by the corner-sharing PbI_6_ octahedra, and I-BA^+^ is the “spacer” that is confined in the interlayer space of the inorganic sheets. Finally, the organic I-BA^+^ cations link with the infinite trilayer *via* N–H⋯I hydrogen bonds to form the 3D network (Fig. S3†). It's worth noting that both confined MA^+^ and I-BA^+^ cations are ordered at room temperature in **IMP**, and are different to MA^+^ cations in the typical 3D perovskites that are highly disordered and random in the cavities. Besides, the configuration of PbI_6_ octahedra is distorted, inferred from the inhomogeneous Pb–I bond lengths (3.0490–3.3334 Å) and I–Pb–I bond angles (86.12–95.19°), as shown in Tables S2 and S3.† As a result, the combination of ordered organic cations and distorted PbI_6_ octahedra leads to the polar structure for **IMP**. The molecular dipole moment and spontaneous polarization were calculated by using the point electric charge model.^38,39^ The spontaneous polarization of **IMP** is estimated to be 1.51 μC cm^−2^ along the *a*-axis and 3.62 μC cm^−2^ along the *c*-axis, respectively (ESI†).

### Semiconducting performance

**Fig. 2:**
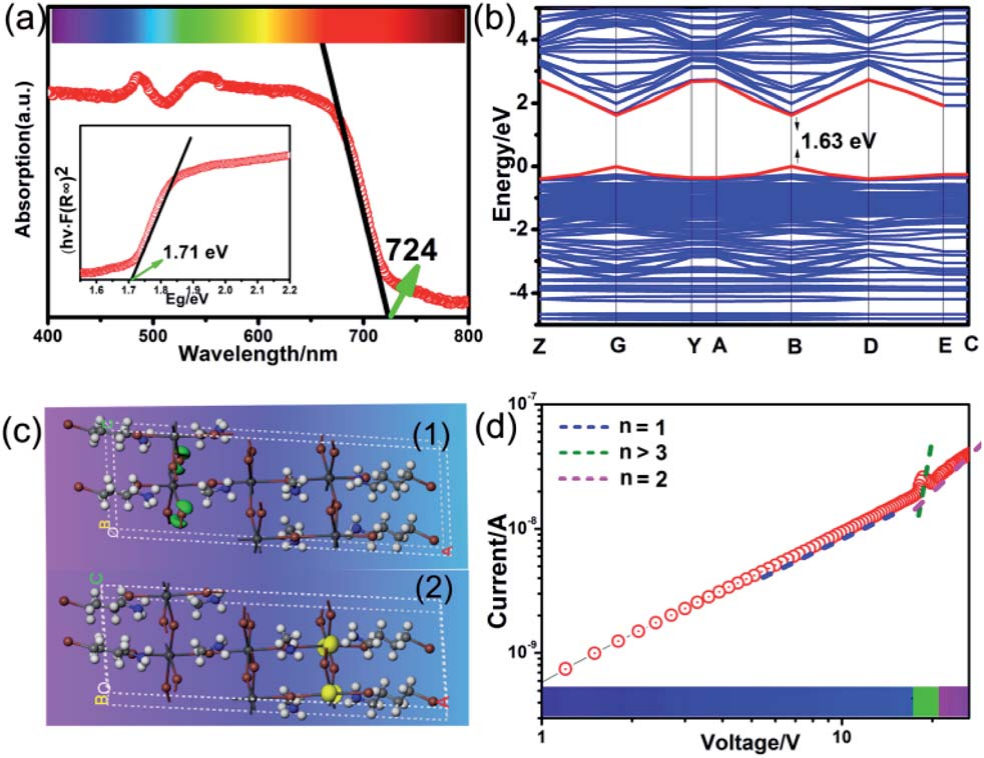
(a) UV-Vis absorption spectrum for **IMP**. Inset: calculated bandgap of **IMP**. (b) Theoretically calculated band structure of **IMP**. (c) The calculated charge density isosurfaces for HOMO (1) and LUMO (2) orbitals. (d) Logarithmic *I*–*V* characteristics of **IMP** based on the space charge limited current method.

The above trilayered perovskite is expected to present striking photoelectric performance benefitting from the transport of photo-excited carriers in the inorganic layer.^40,41^ Large single crystals with sizes up to 9 × 3 × 1.5 mm^3^ can be obtained (Fig. 1c), and the crystal morphology is consistent with the simulated result (Fig. 1b). To evaluate the potential of the **IMP** single crystal for photodetection, its optical and semiconducting properties were studied. Firstly, solid-state ultraviolet-visible (UV-Vis) diffuse reflectance spectroscopy was performed at room temperature. As shown in Fig. 2a, the UV-Vis absorption spectrum of **IMP** displays a broad absorption cutoff at 724 nm, corresponding to a narrow bandgap of 1.71 eV, which is obviously smaller than those of (*S*/*R*-MPA)_2_(MA)Pb_2_I_7_ (2.08 eV) and (4,4-difluorocyclohexylammnium)_2_PbI_4_ (2.38 eV), demonstrating its potential in broad-spectrum self-powered photodetection. Meanwhile, the electronic structure of **IMP** was studied *via* first-principles density functional theory (DFT). As shown in Fig. 2b, both the conduction band minimum and the valence band maximum are localized at the B point, indicating a direct bandgap feature of **IMP**. The calculated bandgap value of 1.63 eV is slightly smaller than the experimental value (1.71 eV), due to the limitation of the DFT methods.^42^ The partial density of states of **IMP** was further analyzed, and it is found that the Pb-6p orbit offers the conduction band minimum while the inorganic framework I-5p orbit dominates the valence band maximum (Fig. S4†). Hence, it is proposed that the inorganic framework determines the optical bandgap and energy structure of **IMP**, consistent with the highest occupied molecular orbital (HOMO) and lowest unoccupied molecular orbital (LUMO). As revealed in Fig. 2c, electrons in the highest occupied molecular orbital (HOMO) are almost around the I atom, while those in the LUMO mainly disperse around the Pb atom. Besides, temperature-dependent conductivity of **IMP** further confirms its typical semiconducting characteristic (Fig. S5†). Finally, the charge transport properties of **IMP** were investigated based on the space charge limited current method (Fig. 2d).^43,44^ The trap density (*n*_traps_) was calculated to be 5.21 × 10^10^ cm^−3^ according to *n*_traps_ = 2*εε*_0_*V*_TFL_/*ed*^2^. Such value is much smaller than those of traditional inorganic semiconductors (CdTe: 10^11^ to 10^13^ cm^−3^,^45^ Si: 10^13^ to 10^14^ cm^−3^,^46^*etc.*), and is comparable to that of reported high-quality MAPbI_3_ (*n*_traps_ = 3.3 × 10^10^ cm^−3^),^47^ demonstrating its potential for high-performance photoresponse.

### Photovoltaic performance and photodetection

**Fig. 3:**
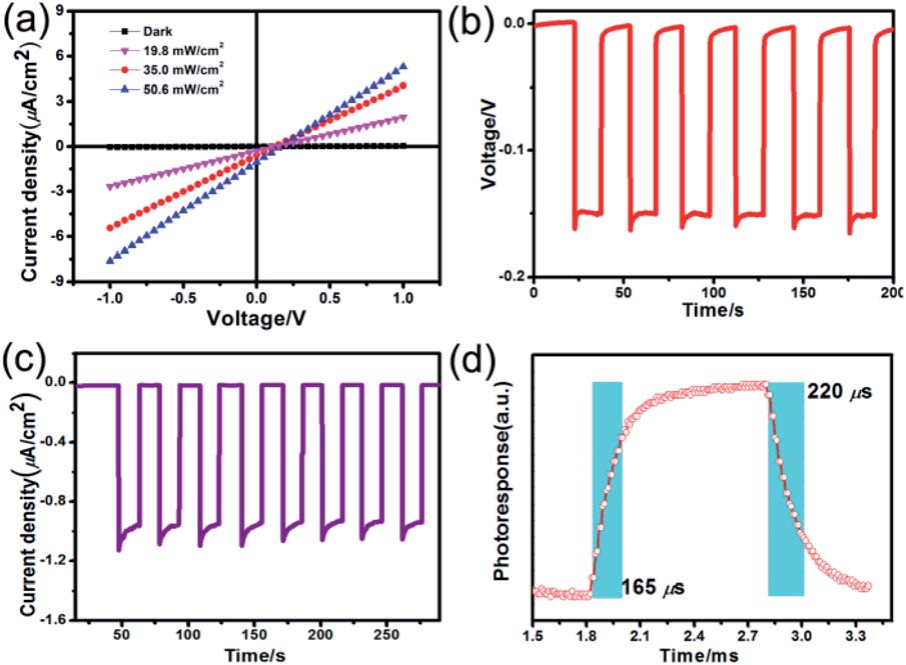
(a) Current–voltage characteristics along polarization directions of **IMP** with different incident power (with a 637 nm laser). (b) Reproducible bulk photovoltaic on/off switching. (c) Reproducible photocurrent on/off switching at zero bias. (d) The rise and fall of the photocurrent at zero bias.

The voltage dependent current (*I*–*V* curve) was measured on high-quality single crystals of **IMP**. The electrodes were attached to the [001] direction of the single crystal, paralleling the polarization orientation of the *c*-axis. Strikingly, the BPVE was clearly observed under irradiation at 637 nm (Fig. 3a). Moreover, the photocurrent density increases directly with the increase of incident light intensity, originating from the enhanced photo-induced carriers. Under an incident light intensity of 50.6 mW cm^−2^, an open-circuit photovoltage of ∼0.15 V is acquired (Fig. 3b), and a spontaneous short-circuit photocurrent reaching up to 1.0 μA cm^−2^ is generated (Fig. 3c). This short-circuit photocurrent is higher than that of reported active self-powered photodetectors, such as BiFeO_3_ (0.4 μA cm^−2^)^48^ and (Pb,La)(Zr,Ti)O_3_ (∼4.0 nA cm^−2^).^17^ Furthermore, the time-dependent photocurrent response of the single-crystal photodetector shows no obvious attenuation after multiple cycles, indicating that the BPV in **IMP** is stable under illumination to realize high-performance self-powered photodetection. Taking advantage of the low trap density in the high-quality single crystal, the **IMP** device exhibits extremely low dark current (∼4 × 10^−12^ A), leading to a high “on/off” ratio of ∼10^3^ under illumination (50.6 mW cm^−2^), and the detectivity is estimated to be 1.25 × 10^9^ Jones by formula (1).1
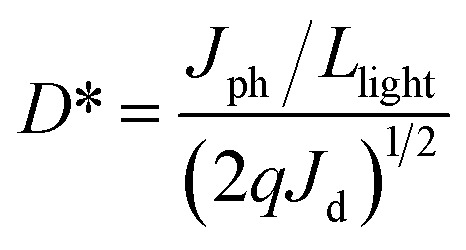
where *J*_ph_ is the photocurrent, *J*_d_ is the dark current, *L*_light_ is the incident light intensity, and *q* is the elementary charge. The time-resolved photoresponse of **IMP** was studied at zero bias. As shown in Fig. 3d, the rise time and fall time were found to be 165 μs and 220 μs (Fig. 3d), which are shorter than those of other reported self-powered photodetectors, such as 0.2 s for EA_4_Pb_3_Cl_10_ and 0.25 s for ZnS,^49,50^ demonstrating the great potential of **IMP** for future high-speed self-powered detection. In addition, considering the broad-spectrum absorption of **IMP**, the photoelectric performances under 405 nm, 520 nm and 700 nm were further investigated. The BPVEs were also observed with notable short-circuit photocurrent (Fig. S6 and S7†). These results indicate that **IMP** is highly promising in broad-spectrum self-powered photodetection.

### Photovoltaic mechanism

**Fig. 4:**
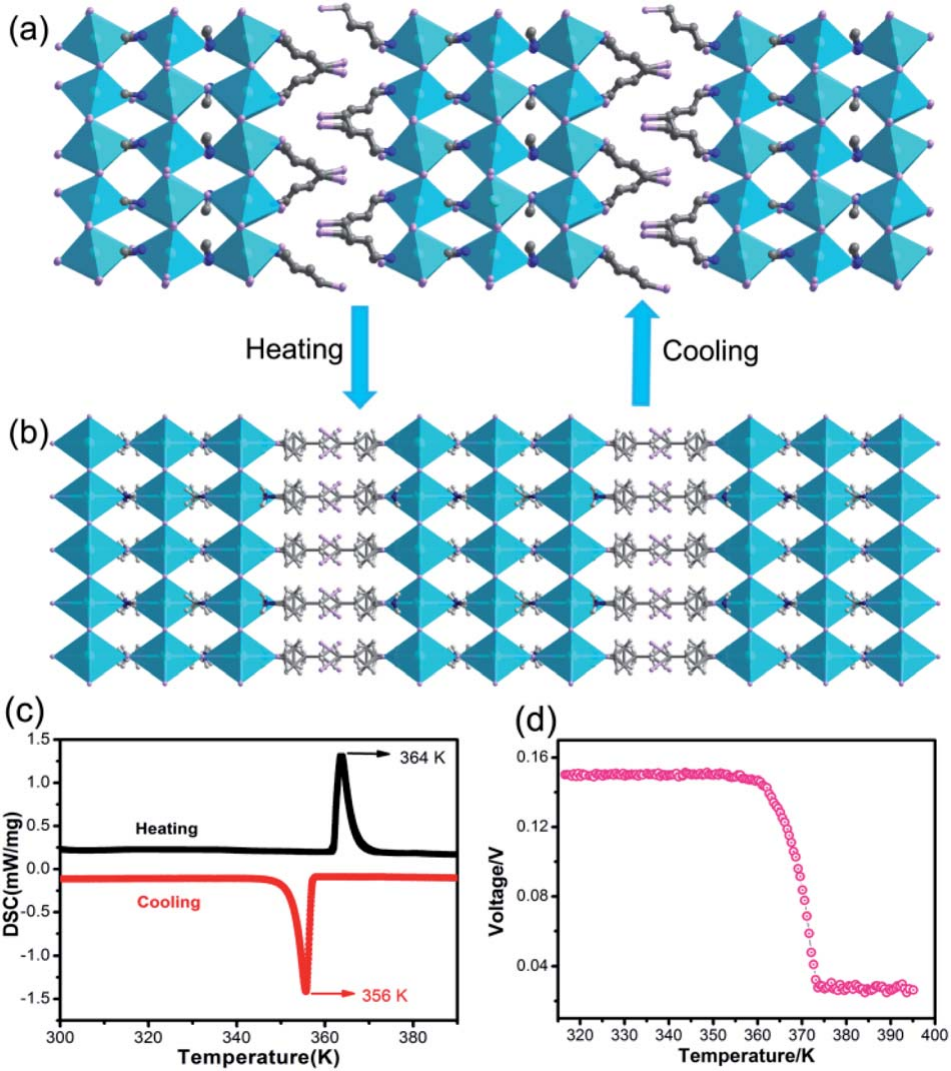
(a) Structure of **IMP** at 290 K. (b) Structure of **IMP** at 385 K. (c) DSC curves in heating and cooling runs for **IMP**. (d) Temperature-dependent photovoltage of **IMP**.

To reveal the relationship between BPVEs and polar structure, variable-temperature structures of **IMP** were analysed. It is found that **IMP** crystallizes in a centrosymmetric space group of *C*2/*m* at 385 K, indicating a structural phase transition accompanying the symmetry breaking during temperature increase (Fig. 4a and b). Variable-temperature PXRDs of the sample were further performed to confirm the reversible phase transition. As shown in Fig. S8,† some PXRD patterns change obviously accompanying the structural phase transition after heating to 395 K, which recover after cooling to room temperature. The presence of a pair of thermal peaks at temperatures of 356 K and 364 K during differential scanning calorimetry measurements further confirms the phase transition (Fig. 4c). Most importantly, the temperature-dependent photovoltage shows a gradual decline with increasing temperature, and disappears completely above 364 K, revealing that the BPVEs in **IMP** depend on the spontaneous polarization in the structure at room temperature (Fig. 4d). At 385 K, the organic cations become disordered and the inorganic layers exhibit a highly symmetric configuration, which endow **IMP** with a centrosymmetric space group without spontaneous polarization. In order to further confirm that the BPVEs originate from spontaneous polarization, the BPVEs along the *a*-axis and *b*-axis were also studied (Fig. S9†). As expected, an obvious BPVE was observed along the *a*-axis, while no signal appeared along the *b*-axis with a gliding plane.

## Conclusions

In summary, we developed a polar trilayered hybrid perovskite *via* dimension reduction of 3D MAPbI_3_. This I-based trilayered hybrid perovskite enables a broad spectrum absorption extending to 724 nm, corresponding to a narrow optical bandgap of ∼1.71 eV. Strikingly, benefiting from the intrinsic polar feature, BPVE was acquired with an open-circuit voltage of 0.15 V and a short-circuit current density of 1.0 μA cm^−2^ under 637 nm illumination. Moreover, the high-quality crystal device of **IMP** exhibits a high “on/off” ratio of ∼10^3^ and photodetectivity (∼10^9^ Jones) at zero bias. These features make **IMP** a promising candidate for the self-powered broad-spectrum detection. This work provides a foothold for developing high-performance self-powered photodetectors in emerging application fields.

## Author contributions

D. Li prepared the samples and wrote the manuscript. W. T. Wu and S. G. Han carried out the structure characterization. X. T. Liu and D. Li analyzed the photoelectric properties. Y. Peng, X. Q. Li, M. C. Hong and J. H. Luo provided suggestions for the project. L. N. Li designed and directed this project. All the authors discussed and commented on the manuscript.

## Conflicts of interest

There are no conflicts to declare.

## Supplementary Material

SC-012-D0SC06112C-s001

SC-012-D0SC06112C-s002
