# Supplementary material for: A reduced-dimensional polar hybrid perovskite for self-powered broad-spectrum photodetection
Source: Chem Sci. 2021 Jan 4;12(8):3050–4. doi: 10.1039/d0sc06112c (PMC8179401; doi:10.1039/d0sc06112c)
Supplement: SC-012-D0SC06112C-s001 [file SC-012-D0SC06112C-s001.pdf]

Electronic Supplementary Material (ESI) for Chemical Science.
This journal is © The Royal Society of Chemistry 2021

# Supporting Information

## Reduced-dimensional polar hybrid perovskite for self-powered broad-spectrum photodetection

Dong Li,[a, b] Wentao Wu,[a, b] Shiguo Han,[a, b, c] Xitao Liu,[a, b, c] Yu Peng,[a, b] Xiaoqi Li,[a, b] Lina Li,*[a, b, c] Maochun Hong,[a, b, c] Junhua Luo[a, b, c]

[a] State Key Laboratory of Structural Chemistry, Fujian Institute of Research on the Structure of Matter, Chinese Academy of Sciences, Fuzhou, Fujian 350002, China
[b] Fujian Science & Technology Innovation Laboratory for Optoelectronic Information of China, Fuzhou, Fujian 350108, P. R. China
[c] University of Chinese Academy of Sciences, Beijing, 100049, China

*To whom correspondence should be addressed.
lilina@fjirsm.ac.cn

## Experimental Section

**Materials**: $Pb(Ac)_2$ (99%, Aladdin), methylamine (MA, 29%, Aladdin), hydroiodic acid (HI, 48%, Aladdin), Aminobutanol (99%, Aladdin). All the chemicals were bought and used without further purification.

**Synthesis**. $Pb(Ac)_2$ (2.27 g, 6 mmol) was dissolved in 48% aqueous HI solution (15 mL) by heating to boil under constant magnetic stirring to give a yellowish solution. Subsequent addition of Aminobutanol (0.36 g, 4 mmol) and MA ( 0.45 g, 4 mmol) to the hot solution formed yellowish precipitation, which were dissolved under stirring to afford a red solution. Finally, dark-red crystals were obtained after the solution cooling to room temperature.

**Powder X-ray diffraction**. Powder X-ray diffractions (PXRD) were performed on a Rigaku MiniFlex 600 diffractometer at room temperature. The diffraction patterns were collected in the 2θ range of 5°−50° with a step size of 0.5 °/min. The experimental PXRD patterns match fairly well with the simulated data based on the single-crystal structure, which confirm the pure phase of **IMP**.

**Second harmonic generation (SHG) measurement.** SHG experiments of **IMP** were performed on powder samples using the fundamental laser beam with a low divergence (pulsed Nd:YAG at a wavelength of 1064 nm, 5 ns pulse duration, 1.6 MW peak power, 10 Hz repetition rate). The numerical values of the nonlinear optical coefficients for SHG have been determined by comparison with a $KH_2PO_4$ reference.

**Single-crystal structure determination.** Single-crystal X-ray diffractions for **IMP** were performed on a BRUKER D8 with the Mo Kα radiation. The data were processed by the Crystalclear software package. The structures of **IMP** were solved by direct methods and then refined by the full-matrix least-squares refinements on $F^2$ using SHELXLTL software package. All the non-hydrogen atoms were located from the trial structure and refined anisotropically with SHELXTL using the full-matrix least-squares procedure.

**Optical property measurements.** The UV absorptions in solid state were measured at room temperature on a PE Lambda 900 UV-Visible spectrophotometer.

**Detector performance measurement.** The current-voltage and current-time curves were measured using KEITHLEY in an electromagnetically shielded probe station. The light was generated from LEDs with wavelength of 405 nm, 520 nm, 637 nm (THORLABS, ITC4001).

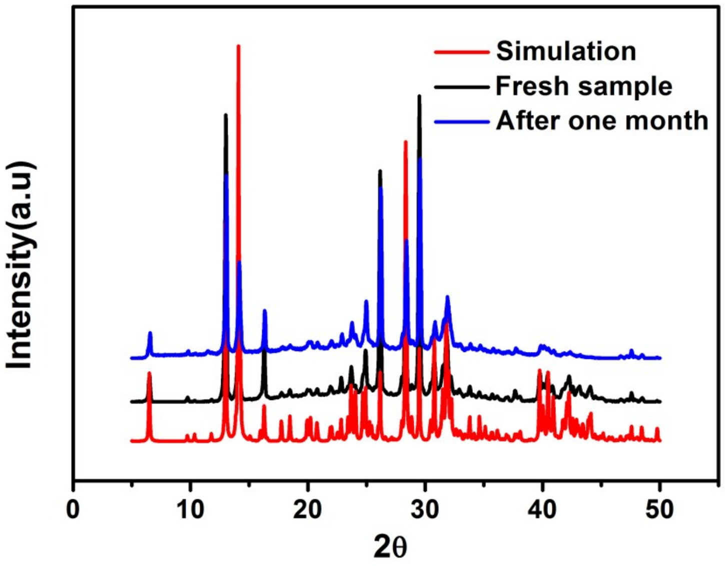


**Figure S1.** X-ray diffraction patterns for **IMP**

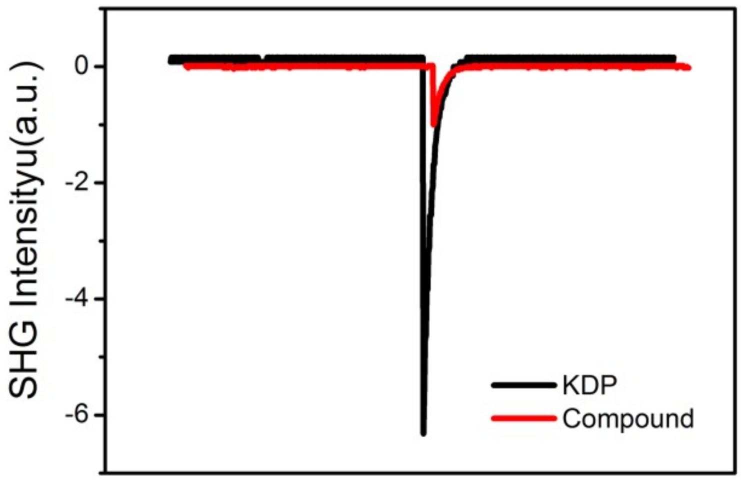


**Figure S2.** The SHG signals for the KDP standard and polycrystalline sample of **IMP** at room temperature.

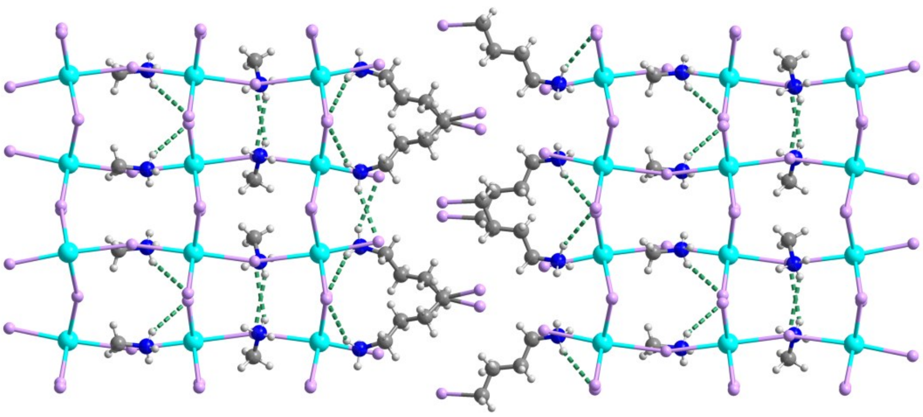

**Figure S3.** Hydrogen bonds in the compound **IMP** (In order to clearly express the hydrogen bond, some hydrogen atoms are removed).

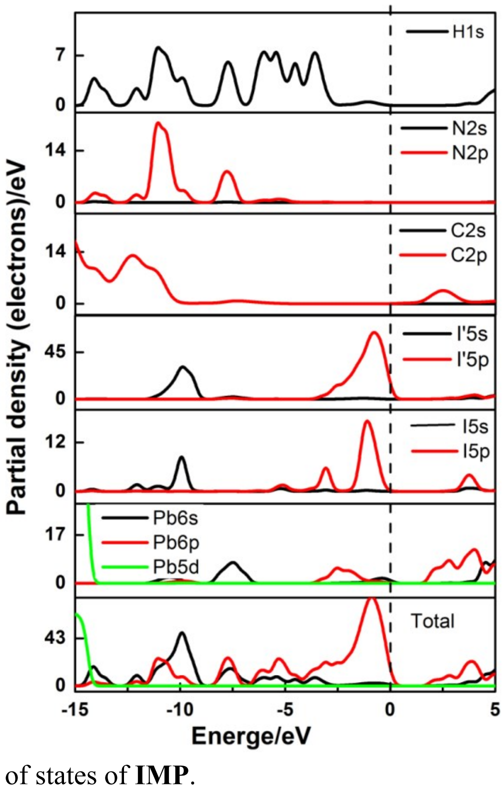


**Figure S4**. Partial density of states of **IMP**.

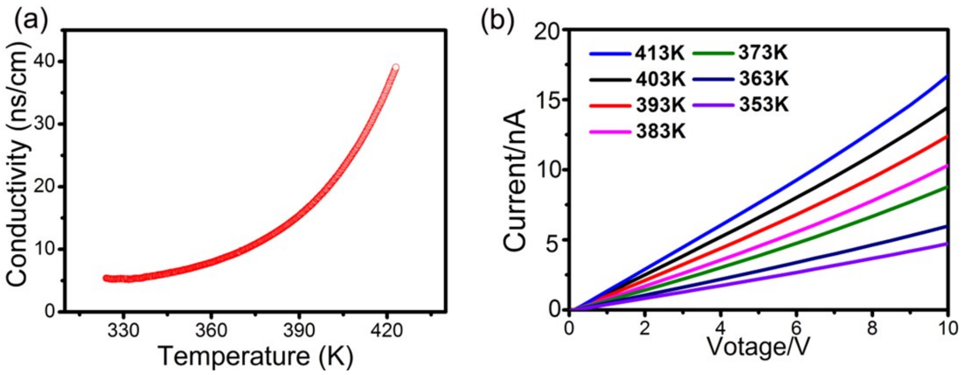


**Figure S5.** (a) Temperature-dependent conductivity. (b) The current-voltage ($I-V$) measurement at various temperatures.

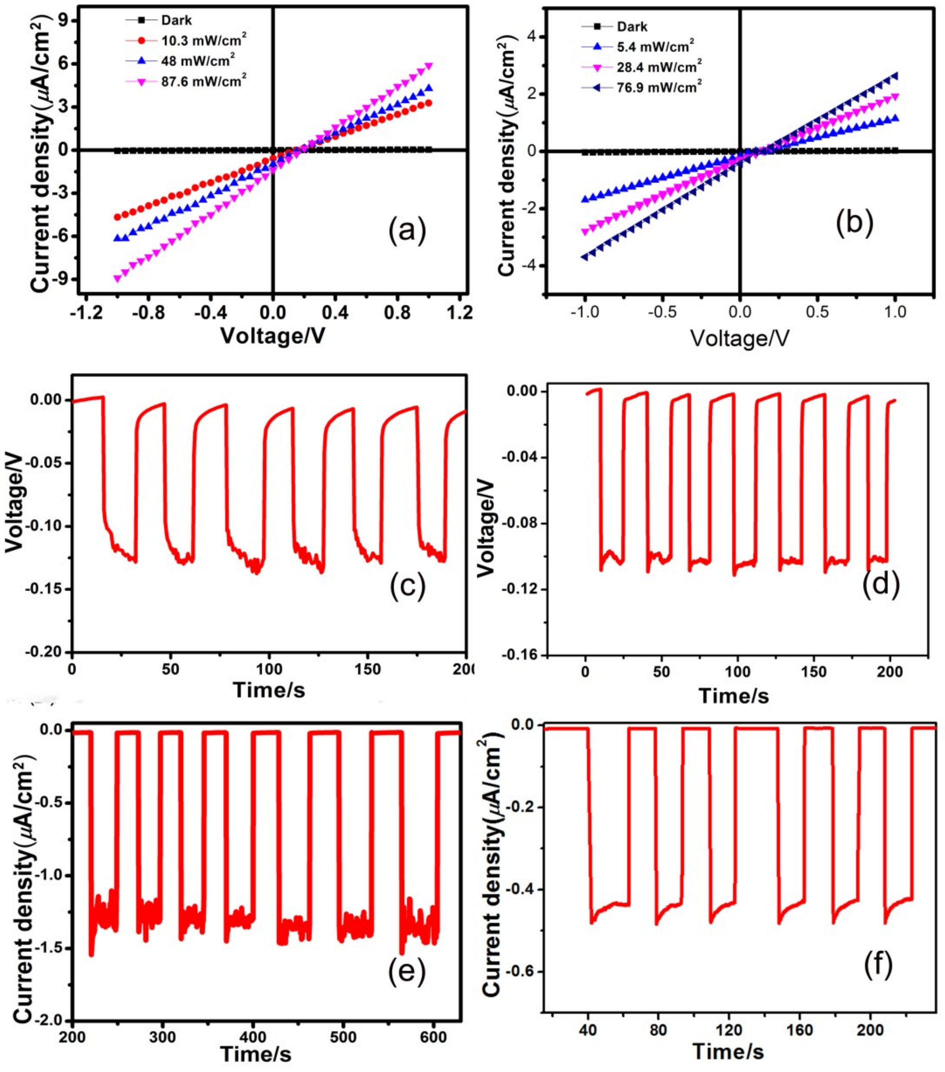


**Figure S6**. (a, b) $I-V$ characteristics along $c$-axis of **IMP** at 520 and 405 nm laser, respectively. (c, d) Reproducible bulk photovoltaic on/off switches of 520 and 405 nm laser. (e, f) Reproducible photocurrent on/off switches of 520 and 405 nm laser at zero bias.

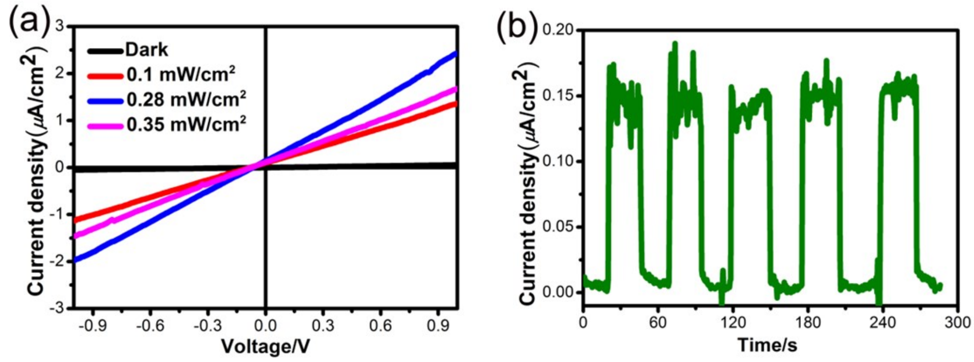


**Figure S7.** (a) *I*−*V* characteristics along *c*-axis for **IMP** at 700 nm. (b) Reproducible photocurrent on/off switches of 700 nm.

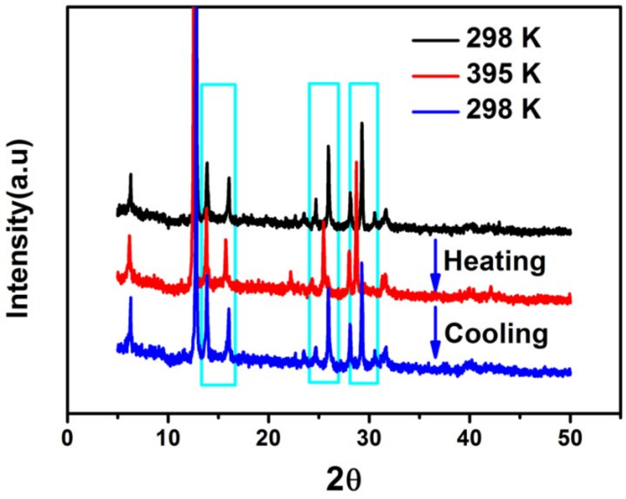


**Figure S8**. PXRD at different temperatures with some changes labelled.

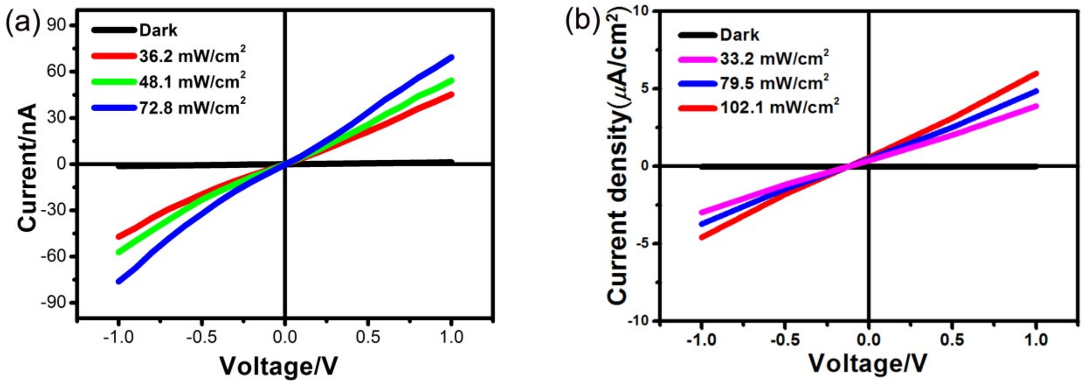


**Figure S9.** (a) *I*−*V* characteristics for *b*-axis of **IMP** with different incident powers. (b) *I*−*V* characteristics for *a*-axis of **IMP** with different incident powers (637 nm laser).

**Table S1.** Crystal data for **IMP**

| Temperature (K) | 290 K | 385 K |
|---|---|---|
| Empirical formula | $C_{10}H_{34}I_{12}N_4Pb_3$ | $C_{10}H_{34}I_{12}N_4Pb_3$ |
| Formula weight | 2354.78 | 2354.78 |
| Temperature/K | 290.0 | 385.0 |
| Crystal system | monoclinic | monoclinic |
| Space group | *Pc* | *C*2/*m* |
| $a$/Å | 27.3848 (12) | 8.909 (2) |
| $b$/Å | 9.0015 (4) | 9.012 (3) |
| $c$/Å | 8.8216 (3) | 27.896 (8) |
| $\alpha$/° | 90 | 90 |
| $\beta$/° | 95.9990 (10) | 92.832 (10) |
| $\gamma$/° | 90 | 90 |
| Volume/Å$^3$ | 2162.65 (15) | 2237.1 (11) |
| Z | 2 | 2 |
| $\rho$calcg/cm$^3$ | 3.616 | 3.496 |
| $\mu$/mm$^{-1}$ | 20.223 | 19.550 |
| F(000) | 2008.0 | 2008.0 |
| Radiation | Mo K$\alpha$ ($\lambda$ = 0.71073) | Mo K$\alpha$ ($\lambda$ = 0.71073) |
| Theta range (°) | 4.486 to 52.84 | 4.386 to 49.982 |
| Index ranges | $-33 \le h \le 34$, $-11 \le k \le 11$, $-11 \le l \le 9$ | $-10 \le h \le 9$, $-10 \le k \le 10$, $-33 \le l \le 33$ |
| Reflections collected | 36105 | 17421 |
| Independent reflections | 8192 [$R_{int}$ = 0.0546, $R_{sigma}$ = 0.0520] | 2111 [$R_{int}$ = 0.0814, $R_{sigma}$ = 0.0495] |
| Data/restraints/parameters | 8192/102/269 | 2111/62/98 |
| GOF | 1.038 | 1.068 |
| Final R indexes [I>=2σ (I)] | $R_1$ = 0.0627, $wR_2$ = 0.1686 | $R_1$ = 0.0948, $wR_2$ = 0.2620 |
| Final R indexes [all data] | $R_1$ = 0.0783, $wR_2$ = 0.1832 | $R_1$ = 0.1099, $wR_2$ = 0.2731 |

**Table S2**. Bond angles for **IMP**.

| Bond | Angle | Bond | Angle |
|---|---|---|---|
| I(1)-Pb(1)-I(3)[#1] | 87.32 (3) | I(5)[#3]-Pb(2)-I(6)[#4] | 88.97(4) |
| I(1)-Pb(1)-I(3) | 93.07(3) | I(5)-Pb(2)-I(6) | 94.33(4) |
| I(1)-Pb(1)-I(2) | 94.06(3) | I(6)-Pb(2)-I(4) | 90.53(5) |
| I(1)-Pb(1)-I(2)[#2] | 88.97(3) | I(6)-Pb(2)-I(5)[#3] | 177.22(4) |
| I(1)-Pb(1)-I(4) | 173.45(3) | I(6)-Pb(2)-I(6)[#4] | 88.305(18) |
| I(3)[#1]-Pb(1)-I(3) | 87.789(11) | I(10)-Pb(3)-I(9) | 93.79(3) |
| I(3)[#1]-Pb(1)-I(2)[#2] | 94.89(3) | I(10)-Pb(3)-I(9)[#4] | 89.85(3) |

| | | | |
|---|---|---|---|
| I(3)[#1]-Pb(1)-I(2) | 176.96(3) | I(10)-Pb(3)-I(7) | 174.65(3) |
| I(3)-Pb(1)-I(4) | 87.26(3) | I(10)-Pb(3)-I(8)[#3] | 87.56(3) |
| I(3)[#1]-Pb(1)-I(4) | 86.16(3) | I(10)-Pb(3)-I(8) | 92.56(3) |
| I(2)[#2]-Pb(1)-I(3) | 176.71(3) | I(9)-Pb(3)-I(9)[#4] | 87.998(11) |
| I(2)-Pb(1)-I(3) | 89.43(3) | I(9)[#4]-Pb(3)-I(7) | 90.68(3) |
| I(2)[#2]-Pb(1)-I(2) | 87.844(11) | I(9)-Pb(3)-I(7) | 91.54(3) |
| I(2)-Pb(1)-I(4) | 92.48(4) | I(9)-Pb(3)-I(8)[#3] | 176.54(3) |
| I(2)[#2]-Pb(1)-I(4) | 91.01(4) | I(9)[#4]-Pb(3)-I(8)[#3] | 95.19(2) |
| I(7)-Pb(2)-I(4) | 179.41(5) | I(8)-Pb(3)-I(9)[#4] | 176.13(3) |
| I(7)-Pb(2)-I(5)[#3] | 89.91(4) | I(8) -Pb(3) -I(9) | 88.82(2) |
| I(7)-Pb(2)-I(5) | 89.98(4) | I(8)[#3] -Pb(3) -I(7) | 87.09(3) |
| I(7)-Pb(2)-I(6) | 89.52(5) | I(8) -Pb(3) -I(7) | 87.20(3) |
| I(7)-Pb(2)-I(6)[#4] | 89.90(5) | I(8) -Pb(3) -I(8)[#3] | 87.939(11) |
| I(4)-Pb(2)-I(6)[#4] | 89.51(5) | Pb(1)[#4] -I(3) -Pb(1) | 163.00(4) |
| I(5)-Pb(2)-I(4) | 90.60(4) | Pb(3) -I(9) -Pb(3)[#1] | 166.78(5) |
| I(5)[#3]-Pb(2)-I(4) | 90.01(4) | Pb(2) -I(7) -Pb(3) | 165.10(5) |
| I(5)-Pb(2)-I(5)[#3] | 88.392(18) | Pb(3) -I(8) -Pb(3)[#2] | 163.66(5) |
| I(5)-Pb(2)-I(6)[#4] | 177.36(4) | Pb(1)[#3]-I(2)-Pb(1) | 164.78(5) |
| Pb(2) -I(4) -Pb(1) | 165.45(6) | Pb(2) -I(5) -Pb(2)[#2] | 167.92(6) |
| Pb(2) -I(6) -Pb(2)[#1] | 165.37(6) | | |

[#1] = +X, 1-Y, 1/2+Z; [#2] = +X, 2-Y, 1/2+Z; [#3] = +X, 2-Y, -1/2+Z; [#4] = +X, 1-Y, -1/2+Z.

**Table S3**. Bond lengths for **IMP**.

| Bond | (Å) | Bond | (Å) | Bond | (Å) |
|---|---|---|---|---|---|
| Pb(1)-I(1) | 3.0634(10) | Pb(2)-I(5)[#3] | 3.1746(13) | Pb(3)-I(8)[#3] | 3.2019(9) |
| Pb(1)-I(3)[#1] | 3.1668(10) | Pb(2)-I(5) | 3.1592(13) | Pb(3)-I(8) | 3.1541(10) |
| Pb(1)-I(3) | 3.1952(10) | Pb(2)-I(6)[#4] | 3.1867(14) | I(3)-Pb (1)[#4] | 3.1668(10) |
| Pb(1)-I(2) | 3.1924(11) | Pb(2)-I(6) | 3.1968(14) | I(9)-Pb(3)[#1] | 3.1905(10) |
| Pb(1)-I(2)[#2] | 3.1749(11) | Pb(3)-I(10) | 3.0490(10) | I(8)-Pb(3)[#2] | 3.2019(9) |
| Pb(1)-I(4) | 3.2623(11) | Pb(3)-I(9) | 3.1639(9) | I(2)-Pb (1)[#3] | 3.1749(11) |
| Pb(2)-I(7) | 3.1434(14) | Pb(3)-I(9)[#4] | 3.1904(10) | I(5)-Pb (2)[#2] | 3.1747(13) |
| Pb(2)-I(4) | 3.1780(14) | Pb(3)-I(7) | 3.3334(10) | I(6)-Pb (2)[#1] | 3.1867(14) |

[#1] = +X, 1-Y, 1/2+Z; [#2] = +X, 2-Y, 1/2+Z; [#3] = +X, 2-Y, -1/2+Z; [#4] = +X, 1-Y, -1/2+Z.

Calculation of polarization of **IMP** according to a point charge model:

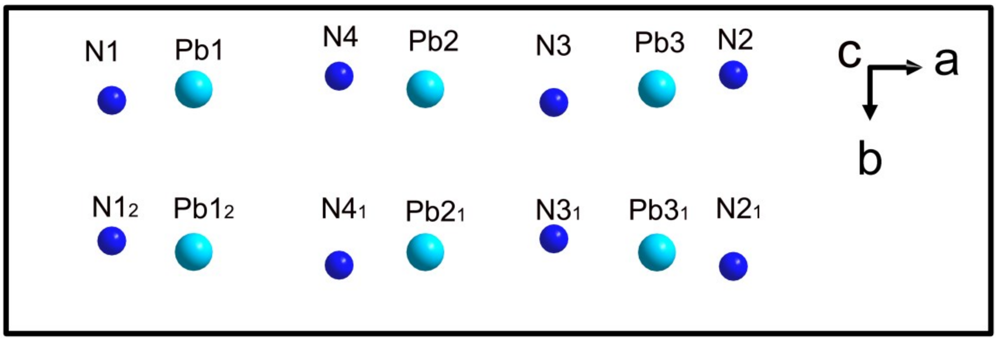


**Figure S10**. Point-charge model for calculating the polarization of **IMP** at 290 K. Hydrogen atoms are omitted for clarity.

Atom coordinates for cell of **IMP** (290 K).

| Atoms | Atoms coordinate | | Coordinate of charge center |
|---|---|---|---|
| Pb | Pb1(0.19055, 0.25067, 0.95588)<br>Pb2 (0.42572, 0.24994, 0.03430)<br>Pb3 (0.66095, 0.24915, 0.11329) | $Pb1_2$ (0.19055, 0.74933, 0.45588)<br>$Pb2_1$ (0.42572, 0.75006, 0.53430)<br>$Pb3_1$ (0.66095, 0.75085, 0.61329) | (0.42574, 0.5, 0.45115) |
| N | N1 (0.1073, 0.28448, 0.3671)<br>N2 (0.73853, 0.2063, 0.6413)<br>N3 (0.55627, 0.29089, 0.538)<br>N4 (0.33825, 0.21002, 0.5366) | $N1_2$ (0.1073, 0.71552, 0.8671)<br>$N2_1$ (0.73853, 0.7937, 0.1413)<br>N b2 (0.55627, 0.70911, 0.038)<br>N b4(0.33825, 0.77899, 0.0366) | (0.43509, 0.5, 0.39575) |

$P_{s\ a\text{-axis}} = [(-e \times 0.42574) \times 6{\times}4/3 + (e \times 0.43509) \times 8] \times a / V$
$= [0.0748 \times 1.6 \times 10^{-19} \times 27.3848 \times 10^{-10}\ C\ m]/V$
$= 3.28 \times 10^{-29}\ C\ m / (2162.65 \times 10^{-30}\ m^3)$
$= 1.51 \times 10^{-2}\ C\ m^{-2}$
$|P_s| = 1.51 \times 10^{-2}\ C\ m^{-2} = 1.51\ \mu C\ cm^2$

$P_{s\ c\text{-axis}} = [(-e \times 0.45115) \times 8 + (e \times 0.39557) \times 8] \times c / V$
$= [0.5558 \times 1.6 \times 10^{-19} \times 8.8216 \times 10^{-10}\ C\ m]/V$
$= 7.84 \times 10^{-29}\ C\ m / (2162.65 \times 10^{-30}\ m^3)$
$= 3.62 \times 10^{-2}\ C\ m^{-2}$
$|P_s| = 3.62 \times 10^{-2}\ C\ m^{-2} = 3.62\ \mu C\ cm^2$
